# High-Mobility Group Box-1 Induces Proinflammatory Cytokines Production of Kupffer Cells through TLRs-Dependent Signaling Pathway after Burn Injury

**DOI:** 10.1371/journal.pone.0050668

**Published:** 2012-11-27

**Authors:** Xu-Lin Chen, Li Sun, Feng Guo, Fei Wang, Sheng Liu, Xun Liang, Ren-Su Wang, Yong-Jie Wang, Ye-Xiang Sun

**Affiliations:** Department of Burns, The First Affiliated Hospital of Anhui Medical University, Hefei, Anhui, PR China; National Institutes of Health, United States of America

## Abstract

Kupffer cells (KCs) were a significant source of cytokine release during the early stage of severe burns. High mobility group box protein 1 (HMGB1) was recently identified as a new type of proinflammatory cytokine. The ability of HMGB1 to generate inflammatory responses after burn trauma has not been well characterized. KCs were isolated from sham animals and rats with a 30% full-thickness burn, and then were stimulated with increasing concentrations of HMGB1. The levels of Tumor necrosis factor (TNF)-α and interleukin (IL)-1β in culture supernatant were measured by enzyme-linked immunosorbent assay. Northern blot analysis was performed to detect the expressions of TNF-α and IL-1β mRNAs. The activities of p38 MAPK and JNK (by Western blot analysis) as well as NF-κB (by EMSA) in KCs were also examined. As a result, HMGB1 *in vitro* upregulated expressions of TNF-α and IL-1β of KCs in a dose-dependent manner, and HMGB1 promoted KCs from burn rats to produce significantly more TNF-α and IL-1β proteins than those from sham animals. After harvested from burn rats, KCs were pre-incubated with anti-TLR2 or anti-TLR4 antibody prior to HMGB1 administration. HMGB1 exposure not only significantly increased expressions of TNF-α and IL-1β mRNAs in KCs from burn rats, but also enhanced activities of p38 MAPK, JNK and NF-κB. However, these upregulation events were all reduced by pre-incubation with anti-TLR2 or anti-TLR4 antibody. These results indicate that HMGB1 induces proinflammatory cytokines production of KCs after sever burn injury, and this process might be largely dependent on TLRs-dependent MAPKs/NF-κB signal pathway.

## Introduction

Despite advances in burn prevention, treatment, and rehabilitation over the last decades, sepsis and subsequent multiple organ dysfunction syndrome (MODS) which were originated from systemic inflammatory response remain to be the most frequently reported causes of death in the severely burned patients [1], [2]. Being central role in metabolism and host defense mechanisms, the liver is thought to be a major organ responsible for the initiation of multiple organ failure in patients with major burns [3]. Proinflammatory cytokines such as tumor necrosis factor (TNF) -α and interleukin (IL)-1β have been demonstrated to be the two most important cytokines in the early phase of burns and play an important role in producing hepatocelluar dysfunction [4].

Locating in the liver sinusoids, Kupffer cells (KCs) comprise the largest population of tissue-fixed macrophages in the human organism. Studies have documented that Kupffer cell played a key role in producing the systemic changes in host immune responses, namely through the up-regulation and release of proinflammatory cytokines [5], [6]. Our previous study has demonstrated that Kupffer cell was a significant source of TNF-α and IL-1β release during the early stage of severe burns, and thereby contributed to the liver injury following thermal injury [7].

High-mobility group box 1 (HMGB1), a highly conserved non-histone chromosomal protein, was originally identified as a DNA-binding protein involved in maintenance of nucleosome structure and regulation of gene transcription [8]. Recently, HMGB1 was found to act as a potent proinflammatory cytokine and a “late” mediator that participated in the development of systemic inflammatory response [9]. Addition of purified recombinant HMGB1 to human monocyte cultures significantly stimulated the release of cytokines including TNF-α, IL-1α, IL-1β, IL-6, and IL-8 [10]. HMGB1 can be either passively released from necrotic or damaged cells, or can be actively secreted by monocytes and macrophages under stressful conditions [11]. Recent data demonstrated that levels of HMGB1 increased significantly in plasma after extensive burn injury, which was associated with the development of sepsis and fatal outcome of major burns [12]. However, the role of HMGB1 in the release of proinflammatory cytokines by KCs following thermal injury has not been fully elucidated so far.

Biological effects of extracellular HMGB1 could be mediated by the activation of signaling pathways coupled to toll-like receptor (TLR) 2, TLR4, TLR9, and the receptor for advanced glycation end products (RAGE) [11], [13], [14]. RAGE has been demonstrated to play only a minor role in macrophages activation by HMGB1, whereas signaling through TLRs, especially TLR2 and TLR4, appears to be of much greater importance in the ability of HMGB1 to generate inflammatory responses [13], [15]. TLR4-deficient mice were found to be less prone to liver injury following burn trauma [16] and the expressions of TLR2 and TLR4 increased in rat macrophages after thermal injury [17], [18]. Moreover, TLR2 and TLR4 could trigger intracellular signaling cascades in macrophages involving activation of p38 mitogen-activated protein kinase (MAPK), c-Jun NH(2)-terminal kinase (JNK), and nuclear factor-κB (NF-κB) [19]. Such signaling activation consequently leaded to the release of proinflammatory cytokines in monocytes including TNF-α and IL-1β [20]. Augmented TLR2 and TLR4 reactivities in macrophages have been demonstrated to contribute to the development of heightened systemic inflammation after burn injury [17]. However, there was little information regarding the potential receptors and signaling mechanisms of HMGB1 underlying immunological function of Kupffer cell after burn injury.

Since its crucial roles in pathophysiological process of inflammation, HMGB1 might regulate the proinflammatory cytokines synthesis in KCs after burn injury. Therefore, the aim of this study was to test the hypothesis that HMGB1 could induce KCs to produce proinflammatory cytokines (TNF-α and IL-1β) through TLRs-dependent signaling after severe burn injury. We show that HMGB1 up-regulated TNF-α and IL-1β releases in cultured KCs by burn injury. Neutralizing antibodies to TLR2 and TLR4 suppress HMGB1-induced activation of inflammatory cascades. In addition, we show that the TLR2 and TLR4 play the key roles in the mechanism of HMGB1-induced activations of p38 MAPK, JNK and NF-κB of KCs in burn injury.

## Materials and Methods

### Animals

Healthy adult male Sprague-Dawley rats weighing 200–250 g were used throughout the study. All experimental manipulations were undertaken in accordance with the National Institutes of Health's Guide for the Care and Use of Laboratory Animals, with the approval of the animal experimental ethics committee of Anhui Medical University, China. All animals were acclimatized to their environment for 1 week. Rats were fed a standard animal diet with food and tap water *ad libitum* for the entire study period.

### Burn procedure

The animals were anesthetized with pentobarbital (30 mg/kg) intraperitoneally, the dorsal and lateral surfaces were shaved, and rats were secured in a constructed template device. The surface area of the skin exposed through the template device was immersed in 98°C water for 12 s on the dorsal surface. With the use of this technique, a full-thickness dermal burn comprising 30% total body surface area (TBSA) were obtained. After immersion, the rats were immediately dried to avoid additional injury, and each animal was resuscitated with an intraperitoneal injection of lactated Ringer's solution (30 ml/kg). Sham burn rats were subjected to an identical preparation except that they were immersed in room temperature water and no fluid resuscitation was administered. Twenty-four hours after burn injury (or sham burn), the rats were exsanguinated by cardiac puncture and KCs were harvested for an *in vitro* study of effects of HMGB1 on cytokines production as described later.

Another group of rats (n = 16) were subjected to a 30% full-thickness burn as described above and killed 24 h later for blocking studies. KCs were harvested for analysis of cytokines production, MAPKs, and NF-κB activations as described later.

### KCs isolation and culture

KCs were isolated and cultured as previously described [7]. In brief, the portal vein was cannulated and the liver was perfused with Hank's balanced salt solution without Ca^2+^ or Mg^2+^ (HBSS) for 10 min at 10–15 ml/min at 30°C followed by 0.2% pronase (Sigma, St Louis, MO, USA; 60 ml at 15 ml/min at 30°C) which was discarded. Following this, the liver was perfused with a recirculating solution of 0.05% pronase and 0.05% collagenase (Sigma, St Louis, MO, USA; 60 ml at 10–17 ml/min) at 30°C for 15–30 min or until the liver was digested as judged by softening of the liver parenchyma beneath the capsule. Next, the livers were dissected free, finely diced, and digested for 20 min at 30°C in 100 ml solution containing 0.02% pronase, 0.05% collagenase and 0.005% DNase (Sigma, St Louis, MO, USA). The supernatant was removed and the pellet resuspended in 10 ml Gey's balanced salt solution without Nacl (GBSS). Aliquots (2.5–5 ml) of this cell suspension were added to 5 ml aliquots of the working metrizamide solution. These were carefully overlaid with 3–4 ml GBSS and centrifuged at 1400×g for 30 min without applying the centrifuge break. The nonparenchymal cell-enriched layer observed at the interface between the two layers was carefully harvested using a blunt 19 gauge needle attached to a 5 ml syringe containing 2 ml GBSS. The viability of cells was greater than 95% as determined by trypan blue exclusion. Cells were cultured at a concentration of 1×10^6^ cell/well in RPMI-1640 medium supplemented with 10% fetal calf serum, 10 mM N-(2-hydroxyethyl)piperazine-N′-(2-ethanesulfonic acid) (HEPES) and 50 µg/ml gentamycin at 37°C in a humidified, 5% CO_2_ atmosphere.

To examine the cell-specific effects of HMGB1 on KCs secretion of TNF-α and IL-1β, KCs were harvested from rats 24 h after either burn trauma (n = 8) or sham burn (n = 8). Then, cells were stimulated with HMGB1 (0, 50, 100, or 200 ng/ml) for 48 h and supernatant was collected to measure KCs secretion of TNF-α and IL-1β. Human HMGB1 was obtained from Sigma-Aldrich Inc, which was expressed in Escherichia coli as an N-terminal histidine-tagged protein and purified under native conditions.

For receptor blocking studies, KCs were isolated from additional burn rats 24 h after thermal injury and divided into 4 groups: 1) negative control, KCs were left untreated and cultured for 48 h; 2) HMGB1 stimulation only, KCs were stimulated with 100 ng/ml HMGB1 for 48 h; 3) HMGB1+anti-TLR2 antibody (InvivoGen, San Diego, CA, USA), KCs were pre-incubated (2 h at 37°C) with anti-TLR2 monoclonal antibody (20 µg/ml) and stimulated with 100 ng/ml HMGB1 for 48 h; and 4) HMGB1+ anti-TLR4 antibody (InvivoGen, San Diego, CA, USA), KCs were pre-incubated (2 h at 37°C) with anti-TLR4 monoclonal antibody (20 µg/ml) and stimulated with 100 ng/ml HMGB1 for 48 h. After culture and stimulation, supernatant was removed for analysis of TNF-α and IL-1β and cells were collected.

### Measurement of TNF-α and IL-1β levels in KCs supernatant

The TNF-α and IL-1β levels were measured in KCs supernatant using a “sandwich” enzyme-linked immunosorbent assay (ELISA) with TNF-α and IL-1β ELISA kits for rats (BioSource International Inc., Camarillo, CA, USA), according to the manufacturer's instructions. All samples were run in duplicate and averaged.

### Northern blot for TNF-α and IL-1β mRNA expressions

Total RNA was isolated using Trizol (Invitrogen Life Technologies Inc.) and quantified spectrophotometrically. RNA (10 µg) was electrophoresed, transferred onto a nylon membrane (Hybond-N^+^; Amersham Corp., Arlington Heights, IL, USA). The blots were hybridized separately with (α-^32^P)dCTP (3000 Ci/mmol; Amersham Corp., Arlington Heights, IL, USA)-labeled rat TNF-α, and IL-1β cDNA probes, subsequently stripped, and reprobed with ^32^P-labeled rat glyceraldehyde-phosphate dehydrogenase (GAPDH) cDNA as internal control, as described by Jia [21]. Densitometric analysis was performed using a Phosphor-Imager system.

### Preparation of cytoplasmic and nuclear extracts

The cytoplasmic and nuclear protein extracts from KCs were prepared as described previously [22]. Protein concentrations were measured using the bicinchoninic acid (BCA) protein assay reagent (Pierce Chemical Company, Rockford, IL, USA) with bovine serum albumin as standard, according to the manufacturer's instructions.

### Western blot analysis for p38 MAPK and JNK

Equal amounts of cytoplasmic proteins (50–100 µg) were separated by sodium dodecyl sulfate-polyacrylamide gel electrophoresis (SDS-PAGE) and electrotransferred to a nitrocellulose membrane. After the blocking procedure, the membrane was then immunoblotted overnight at 4°C with the primary antibody for p38, phosphor-p38, JNK, and phosphor-JNK (Santa Cruz Biotechnology, CA, USA), followed by horseradish peroxidase-conjugated secondary antibodies. Immunoreactive bands were detected using an ECL Western blotting detection system (Amersham, Little Chalfont, UK).

### Electrophoretic mobility shift assay (EMSA) for the DNA binding activity of NF-κB

The EMSA method was used to characterize the binding activity of NF-κB in nuclear extracts as described by Muller [23]. Briefly, a double-stranded oligonucleotide probe containing a consensus binding sequence for NF-κB (5′-AGT TGA GGG GAC TTT CCC AGG C-3′) (Promega Corp., Madison, WI, USA) was labeled with γ(^32^P)ATP (3,000 Ci/mmol at 10 mCi/ml; Amersham Corp., Arlington Heights, IL, USA) using T4 polynucleotide kinase (Promega Corp., Madison, WI, USA). Ten micrograms of nuclear protein were incubated in a 15 µl reaction volume containing 10 mM Tris·HCl (pH 7.5), 5×10^4^ counts/min radiolabeled oligonucleotide probe, 2 µg poly(dIdC), 4% glycerol, 1 mM MgCl_2_, 0.5 mM EDTA, 50 mM NaCl, and 0.5 mM dithiothreitol for 20 min at room temperature. DNA-protein complexes were separated in 6% nondenaturing polyacrylamide gel at 90 V for 2–3 h. Gels were dried, exposed to a Hyperfilm (Amersham Corp., Arlington Heights, IL, USA) at −70°C, and scanned using densitometer. Specificity of the DNA-protein complex was confirmed by competition with a 100-fold excess of unlabeled NF-κB.

### Statistical analysis

All data were expressed as mean ± SEM. Statistical comparisons among groups were analyzed using one-way analysis of variance (ANOVA) and Student–Newman–Keuls (SNK)-q test. We considered p<0.05 to be statistically significant.

## Results

### HMGB1 stimulates greater TNF-α and IL-1β productions by KCs from burn rats than those from sham animals

**Figure 1:**
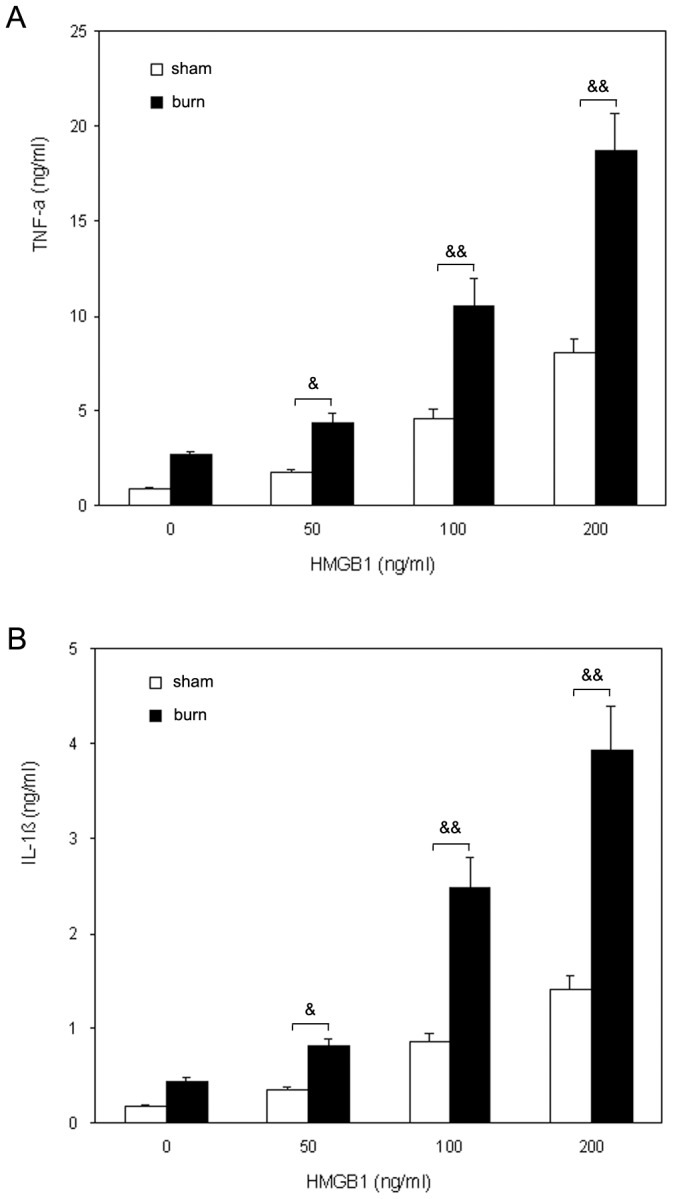
HMGB1 stimulated TNF-α and IL-1β production in KCs from sham and burn rats. KCs responded to an *in vitro* HMGB1 challenge with a significant and dose-dependent increase in TNF-α and IL-1β secretion. HMGB1 induced significantly greater amounts of TNF-α and IL-1β secretion in KCs from burn rats compared to those from sham animals. (A) TNF-α release by KCs from sham and burn rats. (B) IL-1β release by KCs from sham and burn rats. Results were given as mean±SEM (n = 8). & = p<0.05, && = p<0.01 *vs.* related sham group.

KCs from sham and burn rats were treated with various concentrations of HMGB1 (50–200 ng/ml) for 48 h. The TNF-α and IL-1β proteins in supernatant were examined by ELISA. As a result, KCs could produce constitutively few TNF-α and IL-1β under normal condition. HMGB1 stimulated KCs to secrete TNF-α and IL-1β in a dose-dependent manner. Furthermore, HMGB1 induced significantly greater amounts of TNF-α and IL-1β secretion by KCs from burn rats compared to those from sham animals at a dose as low as 50 ng/ml. This discrepancy was even greater when 100 ng/ml or higher concentrations of HMGB1 was used. (Figure 1).

### HMGB1 induces TNF-α and IL-1β synthesis through TLRs signaling in KCs of burn rats

**Figure 2:**
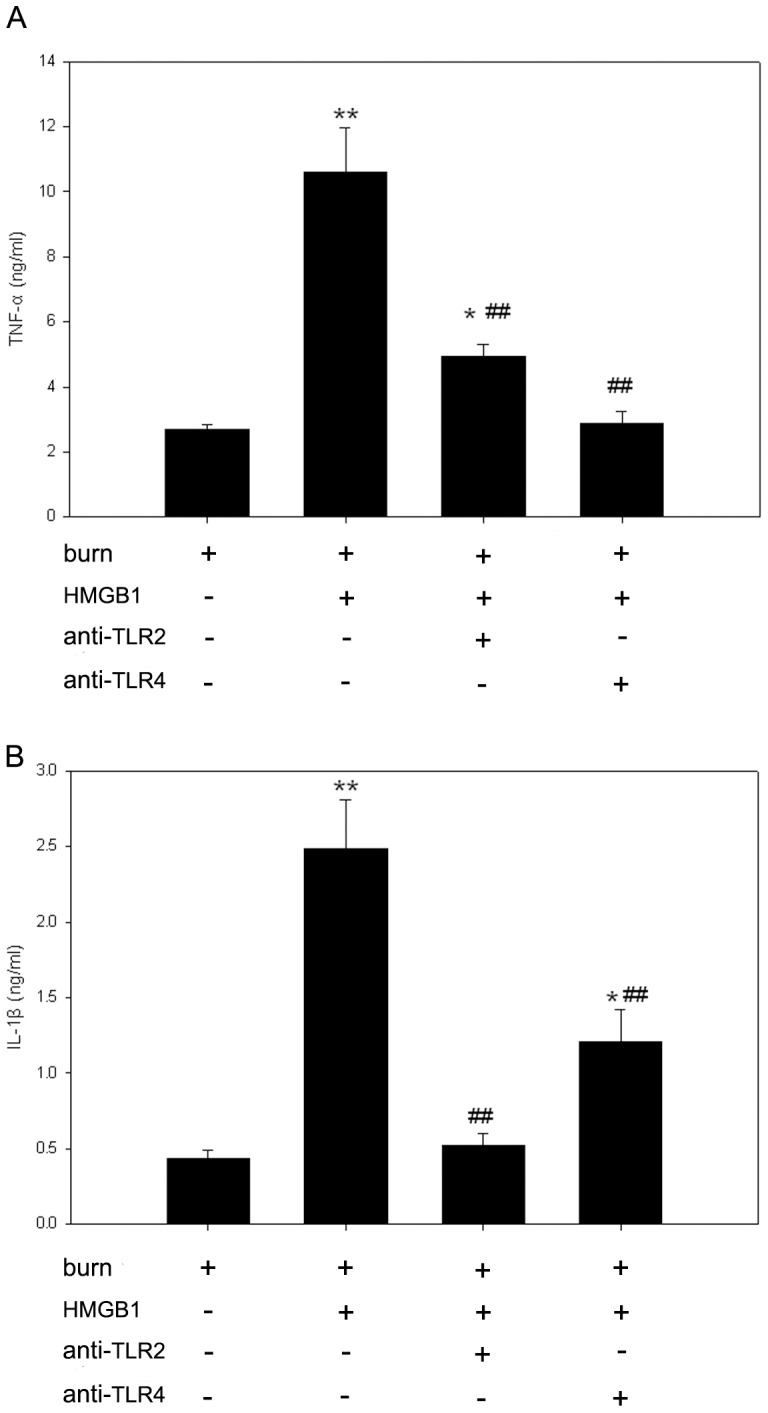
TLRs signaling are required for HMGB1-mediated TNF-α and IL-1β expressions in KCs of burn rats. After 48-h-stimulation with 100 ng/ml HMGB1, KCs released significantly higher levels of TNF-α and IL-1β than those of unstimulated. Pre-incubation of KCs with anti-TLR2 or anti-TLR4 antibody markedly suppressed the release of TNF-α and IL-1β in the HMGB1-stimulated KCs harvested from burn rats. (A) TNF-α release by KCs from burn rats. (B) IL-1β release by KCs from burn rats. Results were given as mean±SEM (n = 8). After burn injury, the KCs treated with medium or HMGB1 alone were used as unstimulated or positive control respectively. * = p<0.05, ** = p<0.01 *vs.* unstimulated controls, ANOVA; ^##^ = p<0.01, *vs.* positive controls, ANOVA.

The productions of TNF-α and IL-1β by KCs following burn trauma were increased significantly 48 h after 100 ng/ml HMGB1 stimulation. Therefore, subsequent analyses on the mechanisms of cell activation were performed with 100 ng/ml HMGB1-stimulated KCs harvested from burn rats. To investigate whether TLRs signaling involve in HMGB1-induced expressions of TNF-α and IL-1β in KCs, anti-TLR2 and anti-TLR4 antibodies were used to treat KCs prior to HMGB1 stimulation (Figure 2). Cells pre-incubated with an isotype control antibody and HMGB1 for 48 h produced similar amounts of TNF-α and IL-1β in response to stimulation as did HMGB1 activated cells in the absence of any antibodies (data not shown).

To characterize the specificity of the TLR2 and TLR4 antibodies, samples incubated with the blocking peptides 2 h before HMGB1 treatment. Immunoblot analysis show levels of TLR2 and TLR4 were blocked by peptide competition (data not shown). Pre-incubation of KCs with anti-TLR2 or anti-TLR4 antibody both significantly attenuated HMGB1-induced TNF-α and IL-1β releases (p<0.01). Anti-TLR4 antibody has more dramatic inhibitory effect than anti-TLR2 antibody on HMGB1-induced TNF-α production (73% *vs.* 53%, p<0.05) and the level of TNF-α in the supernatant of KCs of HMGB1+anti-TLR4 group was similar to that found in unstimulated KCs from burn animals (p>0.05). Meanwhile, TLR2 blockade caused more dramatic inhibition of HMGB1-induced IL-1β production compared to that caused by TLR4 blockade (80% *vs.* 50%, p<0.05) and the level of IL-1β in the supernatant of KCs of HMGB1+anti-TLR2 group was similar to that found in the cells absent of HMGB1 (p>0.05).

**Figure 3:**
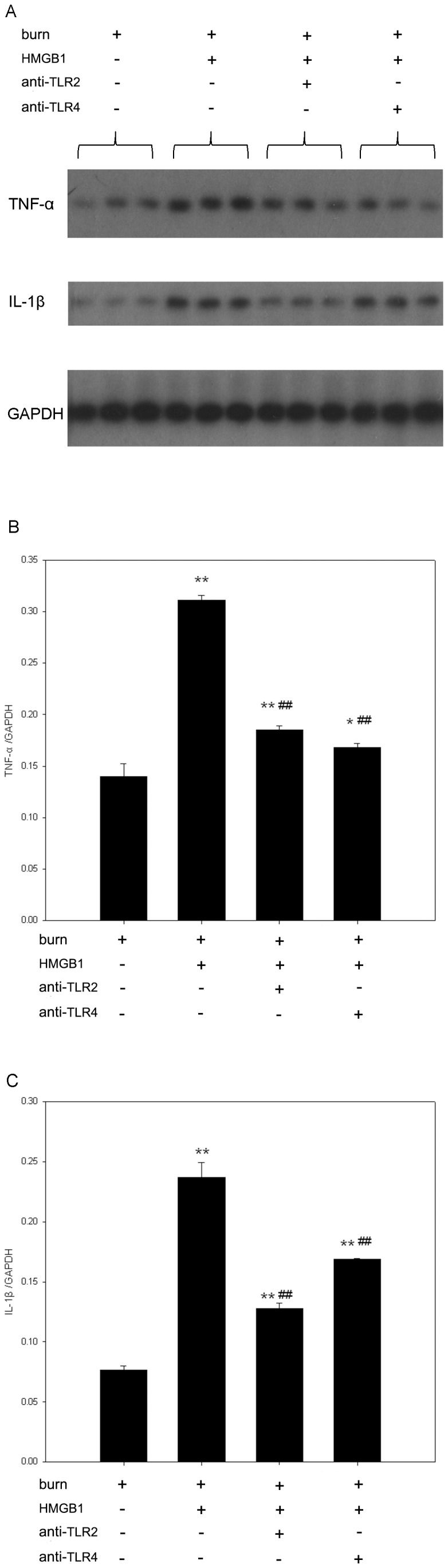
TLR2 and TLR4 involve in HMGB1-induced expressions of TNF-α and IL-1β mRNAs in KCs from burn rats. Expressions of TNF-α and IL-1β mRNAs were measured by Northern blot analysis. Relative mRNA levels were quantified by densitometry and expressed as OD ratio with GAPDH served as internal standards. HMGB1 showed a stimulatory effect on TNF-α and IL-1β mRNA levels in KCs. Pre-incubation of KCs with anti-TLR2 or anti-TLR4 antibody significantly attenuated HMGB1-induced TNF-α and IL-1β mRNA expressions in KCs from burn rats. (A) A representative result. (B) Results of TNF-α mRNA expression from the independent experiments. (C) Results of IL-1β mRNA expression from the independent experiments. Results were given as mean±SEM (n = 3). After burn injury, the KCs treated with medium or HMGB1 alone were used as unstimulated or positive control respectively. ** = p<0.01 *vs.* unstimulated controls, ANOVA; ^##^ = p<0.01, *vs.* positive controls, ANOVA.

In addition, Northern blot analysis of Kupffer cell RNA revealed that TNF-α and IL-1β mRNA levels were significantly augmented by 48-h-stimulation with HMGB1 in comparison with unstimulated controls as corrected by GAPDH transcripts (Figure 3). Moreover, pre-incubation of anti-TLR2 and anti-TLR4 antibodies both significantly inhibited HMGB1-induced expressions of TNF-α and IL-1β mRNAs in KCs of burn rats (p<0.01).

### HMGB1 activates p38, JNK and NF-κB through TLR2 and TLR4 signaling pathways

**Figure 4:**
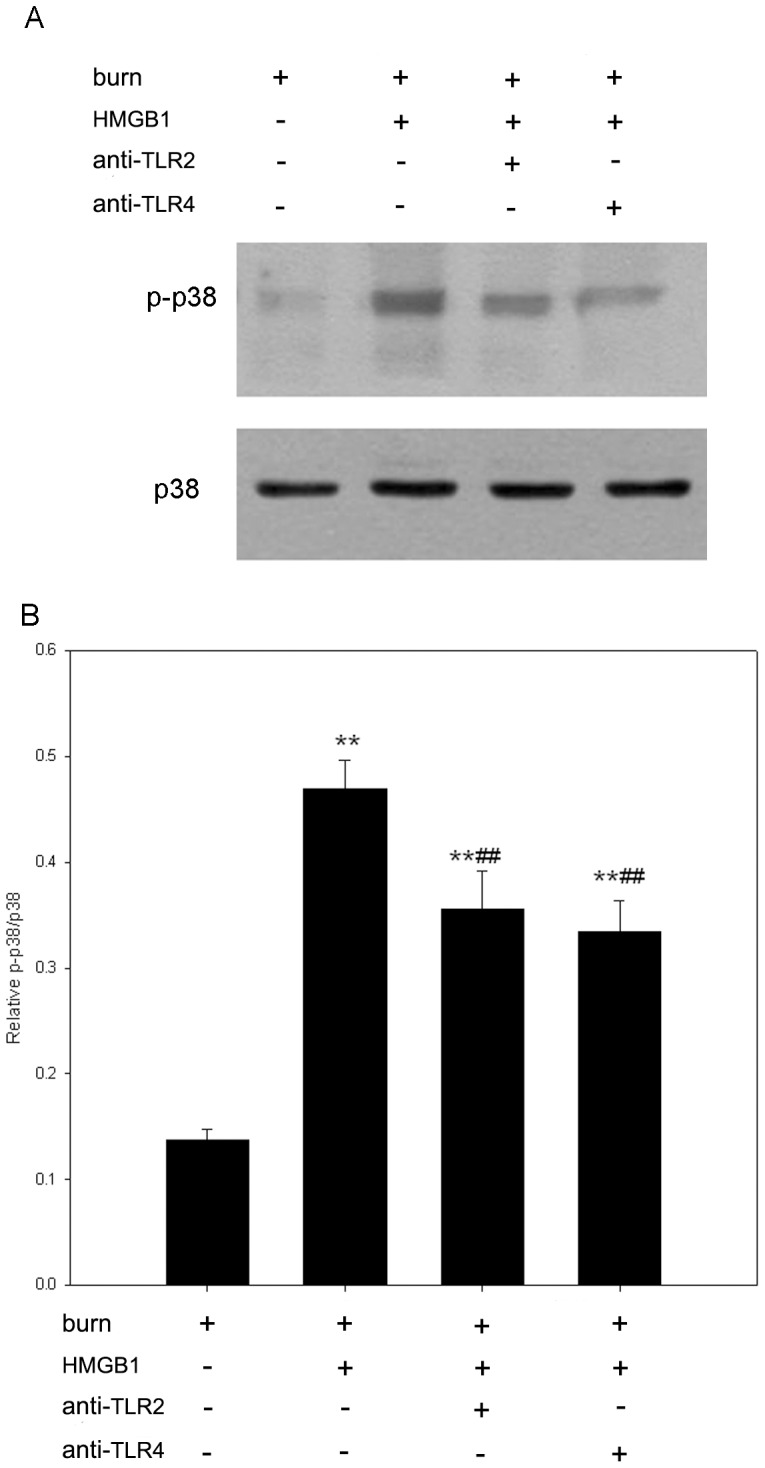
HMGB1 mediates activation of p38 MAPK through TLR2 and TLR4 signalings in KCs from burn rats. After harvested from burn rats, KCs were pre-incubated with anti-TLR2 or anti-TLR4 antibody prior to HMGB1 administration (100 ng/ml, 48 h). Cytosolic extracts were immunoblotted with phospho-p38 and p38 antibodies. The HMGB1-mediated p38 MAPK activation was significantly blocked by pre-incubation with anti-TLR2 or anti-TLR4 antibody in KCs of burn rats. (A) A representative result. (B) Results from the independent experiments. The p38 activity was expressed as relative densitometry of p-p38/p38. Results were given as mean±SEM (n = 8). After burn injury, the KCs treated with medium or HMGB1 alone were used as unstimulated or positive control respectively. ** = p<0.01 *vs.* unstimulated controls, ANOVA; ^##^ = p<0.01, *vs.* positive controls, ANOVA.

**Figure 5:**
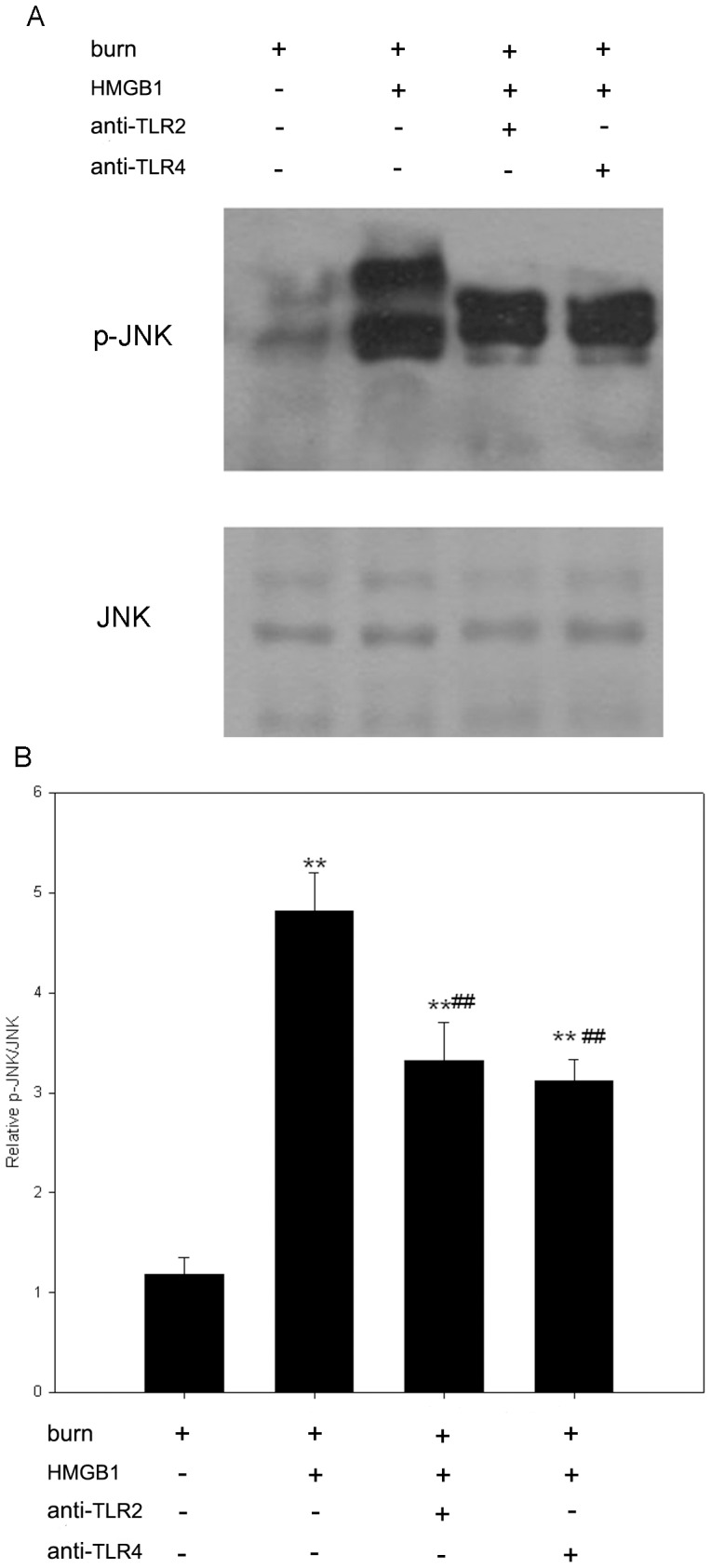
HMGB1 mediates activation of JNK through TLR2 and TLR4 signalings in KCs from burn rats. After harvested from burn rats, KCs were pre-incubated with anti-TLR2 or anti-TLR4 antibody prior to HMGB1 stimulation (100 ng/ml, 48 hrs). Cytosolic extracts were immunoblotted with phospho-JNK and JNK antibodies. The HMGB1-mediated JNK activation was significantly blocked by pre-incubation with anti-TLR2 or anti-TLR4 antibody in KCs of burn rats. (A) A representative result. (B) Results from the independent experiments. JNK activity was expressed as relative densitometry of p-JNK/JNK. Results were given as mean±SEM (n = 8). After burn injury, the KCs treated with medium or HMGB1 alone were used as unstimulated or positive control respectively. ** = p<0.01 *vs.* unstimulated controls, ANOVA; ^##^ = p<0.01, *vs.* positive controls, ANOVA.

Since NF-κB and MAPK play pivotal roles in intracellular TLRs signaling events, and NF-κB has been shown to be involved in regulating the expression of genes that encode inflammatory cytokines, such as TNF-α and IL-1β [24]. We further investigate the effects of HMGB1 on activation of p38, JNK and NF-κB in KCs of burn rats. In the present study, after harvested from burn rats, KCs were pre-incubated with anti-TLR2 or anti-TLR4 antibody prior to HMGB1 administration (100 ng/ml, 48 h). The phosphorylation of p38 and JNK were detected by Western blot assays. As a result, phosphorylation of p38 was significantly increased in HMGB1-stimulated KCs compared to control cells treated with medium only (Figure 4). Anti-TLR2 and anti-TLR4 antibodies both significantly decreased phosphorylation of p38 in HMGB1-stimulated cells, and reductions up to 75% and 71% respectively. There was no significant difference in total p38 expression between groups. Furthermore, HMGB1 also markedly upregulated JNK activity in KCs when compared to unstimulated KCs. Anti-TLR2 and anti-TLR4 antibodies performed the similar inhibitory effects on HMGB1-induced JNK activation as p38 activation in KCs of burn rats. (Figure 5). Total JNK expression was not changed over time.

**Figure 6:**
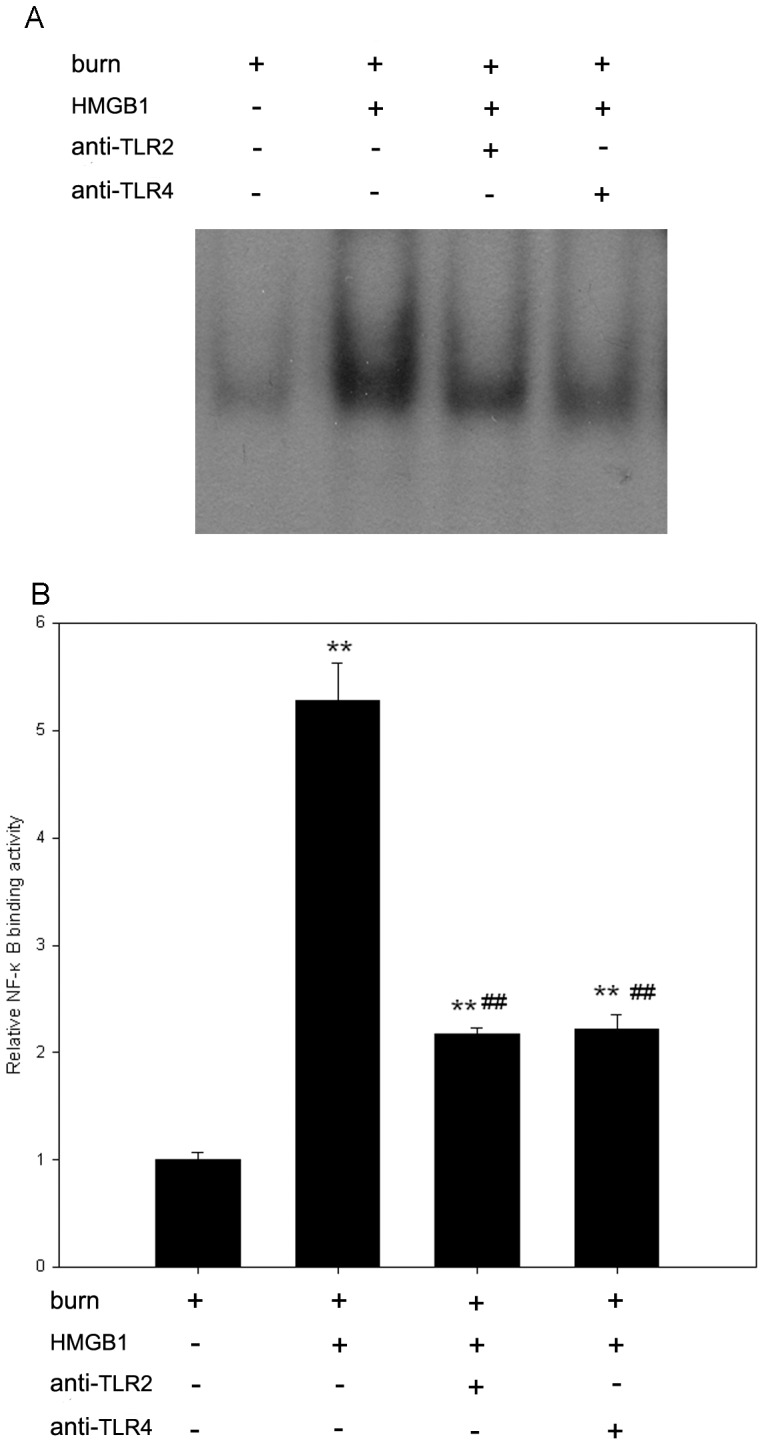
HMGB1 induces activation of NF-κB through TLR2 and TLR4 signalings in KCs from burn rats. KCs were lysed, and nuclear extracts were prepared. Nuclear extracts were incubated with NF-κB-specific radio-labeled oligonucleotides, and detected by EMSA. KCs treated with HMGB1 (100 ng/ml, 48 h) exhibit an increase in NF-κB binding to radio-labeled oligonucleotides, compared with unstimulated cells. However, pre-incubation with anti-TLR2 or anti-TLR4 antibody inhibited HMGB1-induced activation of NF-κB in KCs harvested from burn rats. (A) A representative result. (B) Results from the independent experiments. NF-κB shift bands were quantified by densitometry and expressed as n-fold increases *vs* control values. Results were given as mean±SEM (n = 3). After burn injury, the KCs treated with medium or HMGB1 alone were used as unstimulated or positive control respectively. ** = p<0.01 *vs.* unstimulated controls, ANOVA; ^##^ = p<0.01, *vs.* positive controls, ANOVA.

In the present study, binding activity of nuclear protein to the radiolabeled consensus binding sequences of NF-κB was assessed by EMSA in KCs from burn rats. Forty-eight hours after 100 ng/ml HMGB1 stimulation, NF-κB was significantly activated in KCs in comparison with the unstimulated control (p<0.01). This up-regulation was greatly attenuated in KCs pre-incubated with the anti-TLR2 or anti-TLR4 antibody (p<0.01) (Figure 6).

## Discussion

HMGB1, a 25 KDa nuclear protein, is a late cytokine mediator of lethal endotoxemia and sepsis. After burn injury, HMGB1 is released passively during cell injury, and is actively secreted by macrophages and other cell types activated by exposure to products of thermal injury or infection [25], [26]. It has been implicated as a mediator of tissue damage and inflammation after burn trauma [12], [27], [28]. The delayed kinetics of HMGB1 release makes it an attractive therapeutic target with a wider window of opportunity for the treatment of lethal systemic inflammation [29]. Kupffer cell, continuously exposed to possible blood-borne pathogens from the gastrointestinal tract through the portal vein or from the systemic circulation through the hepatic artery, represents an early line of defense against blood-borne infections [30]. It has been suggested to be the major source of proinflammatory cytokine which mediated deleterious inflammatory injury after burn trauma.

In this study, HMGB1 stimulation to KCs induced the secretion of inflammatory cytokine TNF-α and IL-1β in a dose-dependent manner. Interestingly, the expressions of TNF-α and IL-1β in burn rats seemed to be more sensitive for HMGB1 stimulation than that in non-burn rats. The releases of TNF-α and IL-1β by KCs from severely burned rats were significantly more than that from non-burn animals after HMGB1 treatment, indicating that HMGB1 might play a crucial role in amplifying inflammation cascades after burn injury. Macrophage hyperactivity with increased production of TNF-α, IL-6, IL-1β, and prostaglandins has been demonstrated in the injured patient [31]. Burn injury could induce Kupffer cell in the hyperactivity state [32], which is responsible for stronger responsiveness of KCs to HMGB1 in the present study.

Extensive burn injury could result in significantly increased HMGB1 levels, which appears to be associated with the development of sepsis and fatal outcome of major burn [12], [25]. HMGB1 mRNA expression was significantly increased in liver at 24 h postburn, which remained markedly elevated up to 72 h after thermal injury [33]. In addition, there were high positive correlations between hepatic HMGB1 mRNA and serum aminoleucine transferase (ALT) and aspartate aminotransferase (AST) levels [34]. These results suggested that liver was a source of HMGB1 after burn trauma. In the model of sepsis, extracellular HMGB1 can function as an alarm in signal to recruit, alert and activate KCs and other innate immune cells, thereby sustaining a potentially injurious inflammatory response [34]. Therefore, HMGB1 may activate KCs *in vivo* after burn injury. This merits further studies.

HMGB1 interacts with different receptors in signaling for stimulation of cytokine release in primary cells and established cell lines [29]. Elucidating HMGB1 receptor usage in processes where HMGB1 acts is essential for understanding of basic HMGB1 biology and for designing HMGB1-targeted therapies after burns. Several studies have documented a role of TLR2, TLR4, and RAGE in HMGB1-induced macrophages activation *in vitro*, although some discrepant results were reported [35]. HMGB1 was shown to rapidly interact with TLR2 and TLR4 exposed by RAW macrophage-like cells in one study [36], but not in the other study [29]. RAGE has been demonstrated to play only a minor role in macrophage activation by HMGB1 [15], [37]. Our previous study also showed that pre-incubation of KCs with anti-RAGE antibody and recombinant mouse RAGE/Fc chimera both failed to decrease HMGB1-induced TNF-α and IL-1β production [data not shown], implying that the majority of KCs activation by HMGB1 was independent of RAGE. In the present study, HMGB1 not only promoted KCs synthesis of TNF-α and IL-1β proteins but also increased the mRNAs expression after burn trauma. However, HMGB1-induced expressions of TNF-α and IL-1β were significantly inhibited by pre-incubation with anti-TLR2 or anti-TLR4 antibody. These results demonstrate HMGB1-induced KCs production of proinflammatory cytokines after burn injury is mediated via TLRs-dependent processes.

TLRs are a group of highly conserved proteins that activate innate immune cells in response to a variety of endogenous and exogenous stimulus. However, whether there is differential usage of TLR2 and TLR4 in HMGB1 signaling in KCs after burn injury still need further investigation. In the present study, our data that TNF-α production induced by HMGB1 is more sensitive to TLR4 blocking antibody than to TLR2 blocking antibody suggest that TLR4 plays a major role in HMGB1-induced TNF-α production. This result is consistent with studies by van Zoelen and colleagues [36], who reported that HMGB1-stimulated TNF-α from RAW macrophages are dose-dependently inhibited by anti-TLR4 antibodies, but not by anti-TLR2 antibodies and HMGB1 stimulated significantly less TNF-α release observed in TLR4 knockout cells than TLR2 knockout cells. On the other hand, anti-TLR2 antibody has more dramatic inhibitory effect than anti-TLR4 antibody on HMGB1-induced IL-1β production, suggesting HMGB1-induced IL-1β production mainly involving TLR2. These findings highlight the differential usages of TLR2 and TLR4 in HMGB1 signaling in cytokine release of KCs following burn injury, adding complexity to studies of HMGB1 signaling which was not previously expected.

The signaling transduction of TLR2 and TLR4 results in activation of MAPKs and NF-κB, which plays a significant role in the immune cell recruitment and function [30], [37]. MAPKs are a family of serine/threonine protein kinases responsible for most cellular responses to cytokines and external stress signals and crucial for regulation of the production of inflammation mediators [38]. The mammalian family of MAPKs includes extracellular signal-regulated kinase (ERK), p38, and JNK, and ERK5. Persistent activation of the JNK or p38 signaling pathway has been demonstrated to lead to induction of many proteins central to inflammatory process including further induction of cytokine secretion [39]. Our previous study also suggested that activation of p38 MAPK played a pivotal role in the regulation of the KCs production of proinflammatory cytokines following burn trauma [7]. In the present study, we found that HMGB1 increased the activities of p38 MAPK and JNK in KCs after thermal injury and this up-regulation was significantly inhibited by pre-incubation with anti-TLR2 or anti-TLR4 antibody. These findings therefore suggest that the proinflammatory effects of HMGB1 on KCs after burn injury are mediated via TLRs-dependent MAPK signal pathway.

NF-κB, an ubiquitous transcription factor that regulates the expression of various cytokines and their receptors, involved in the pathological responses of inflammation after burn trauma [39]. The gene of proinflammatory cytokines such as TNF-α and IL-1β contains a number of NF-κB-like binding sites within its promoter region [40]. In this study, HMGB1 increased the translocation of NF-κB into nucleus of KCs after burn injury, which could be suppressed by pre-incubation with anti-TLR2 or anti-TLR4 antibody, suggesting that TLRs-triggered activation of NF-κB pathway is responsible for HMGB1-induced cytokines releasing after burn injury.

Recently, p38 MAPK and JNK have been implicated in the activation of NF-κB [41], [42]. Jiang and his coworkers have demonstrated that transfection with p38 MAPK siRNA or the use of p38 MAPK inhibitor SB203580 markedly attenuates LPS-stimulated NF-κB p65 translocation to nucleus and monocyte chemoattractant protein-1 production in rat vascular smooth muscle cells [43]. LPS-induced translocation of NF-κB into nucleus and degradation of IκB-α was blocked by SP600125, a specific inhibitor of JNK, in human tracheal smooth cells [44]. SP600125 has also been found to inhibit the phosphorylation of NF-κB p65 in rat mesangial cells [45]. Thus, it can be postulated that TLRs-dependent activations of p38 MAPK and JNK regulated NF-κB signaling in KCs following burn trauma.

In this study, anti-TLR2/TLR4 neutralization inhibited HMGB1-induced production of TNF-α and IL-1β by about 60∼90%, however, the inhibition of HMGB1-induced activation of MAPK p38, JNK and NF-κB was much less. Except for TLR2/TLR4, RAGE and TLR9 are also the receptors for the proinflammatory activity of HMGB1 in macrophages. HMGB1 has the potential to induce a production of several proinflammatory mediators from rat macrophages via phosphorylation of MAPK and translocation of NF-κB, with RAGE and TLR9 as the activation-inducing receptors [46], [47].The multiple signaling pathways of HMGB1 accounted for the discrepancy of HMGB1-induced production of proinflammatory cytokines and activation of intracellular signals in the present study.

There are several HMGB1 inhibitors such as anti-HMGB1 antibody, recombinant Box-A, glycyrrhizin. In addition with the presented experiments against the putative HMGB1 receptors TLR2 and TLR4, blocking experiments using specific HMGB1 inhibitors (like neutralizing antibody, glycyrrhizin or HMGB1 box A), in our opinion, will strengthen the presented data and awaits the results of further studies.

In summary, the data of this study showed that HMGB1 promoted KCs secretion of inflammatory cytokine TNF-α and IL-1β, up-regulated the activities of p38 MAPK, JNK as well as NF-κB after burn injury, all of which could be suppressed by pre-incubation with anti-TLR2 or anti-TLR4 antibody. These results indicate that HMGB1-induced proinflammatory cytokines of KCs following burn injury are mediated through TLRs-dependent MAPKs/NF-κB signal pathway. Therefore, our findings provide a new insight into the molecular mechanisms accounting for HMGB1 exacerbating inflammatory responses after thermal injury.
